# Vaginal Heparan Sulfate Linked to Neutrophil Dysfunction in the Acute Inflammatory Response Associated with Experimental Vulvovaginal Candidiasis

**DOI:** 10.1128/mBio.00211-17

**Published:** 2017-03-14

**Authors:** Junko Yano, Mairi C. Noverr, Paul L. Fidel

**Affiliations:** a Department of Oral and Craniofacial Biology, Louisiana State University Health Sciences Center School of Dentistry, New Orleans, Louisiana, USA; b Department of Prosthodontics, Louisiana State University Health Sciences Center School of Dentistry, New Orleans, Louisiana, USA; Albert Einstein College of Medicine

**Keywords:** *Candida albicans*, heparan sulfate, polymorphonuclear leukocytes, vulvovaginal candidiasis

## Abstract

Despite acute inflammation by polymorphonuclear neutrophils (PMNs) during vulvovaginal candidiasis (VVC), clearance of *Candida* fails to occur. The purpose of this study was to uncover the mechanism of vaginal PMN dysfunction. Designs included assessing PMN migration, proinflammatory mediators, and tissue damage (by analysis of the activity of lactate dehydrogenase [LDH]) in mice susceptible (C3H/HeN-C57BL/6) or resistant (CD-1) to chronic VVC (CVVC-S or CVVC-R) and testing morphology-specific *Candida albicans* strains under conditions of preinduced PMN migration (CVVC-S mice) or PMN depletion (CVVC-R mice). *In vitro* designs included evaluation of *C. albicans* killing by elicited vaginal or peritoneal PMNs in standard or vaginal conditioned medium (VCM). Results showed that despite significant migration of PMNs and high levels of vaginal beta interleukin-1 (IL-1β) and alarmin S100A8, CVVC-S mice failed to reduce vaginal fungal burden irrespective of morphology or whether PMNs were present pre- or postinoculation, and had high LDH levels. In contrast, CVVC-R mice had reduced fungal burden and low LDH levels following PMN recruitment and IL-1β/S100A8 production, but maintained colonization in the absence of PMNs. Elicited vaginal and peritoneal PMNs showed substantial killing activity in standard media or VCM from CVVC-R mice but not in VCM from CVVC-S mice. The inhibitory effect of VCM from CVVC-S mice was unaffected by endogenous or exogenous estrogen and was ablated following depletion/neutralization of Mac-1 ligands using Mac-1^+/+^ PMNs or recombinant Mac-1. Heparan sulfate (HS) was identified as the putative inhibitor as evidenced by the rescue of PMN killing following heparanase treatment of VCM, as well as by inhibition of killing by purified HS. These results suggest that vaginal HS is linked to PMN dysfunction in CVVC-S mice as a competitive ligand for Mac-1.

## IMPORTANCE

Vaginal candidiasis, caused by *Candida albicans*, affects a significant number of women worldwide. Despite an acute inflammatory response by neutrophils during infection, the response fails to reduce the organism. Instead, the response is considered a key process underlying the symptoms of vaginitis. Therefore, it is important to determine the mechanism(s) associated with the lack of vaginal neutrophil antifungal activity. The established mouse model of *Candida* vaginitis was used to uncover the mechanism of neutrophil dysfunction. Results revealed that heparan sulfate present in the vagina of mice susceptible to chronic vaginitis served as a competitive ligand for the receptor (Mac-1) necessary for fungal recognition and neutrophil-mediated killing. This inhibitory function of heparan sulfate, confirmed through several approaches, provides the first evidence to explain the lack of antifungal immune reactivity during vaginal candidiasis. This finding paves the way for design of therapeutic strategies to reduce/eliminate symptomatic vaginal candidiasis and restore quality of life to those affected.

## INTRODUCTION

Vulvovaginal candidiasis (VVC) is an opportunistic fungal infection caused primarily by *Candida albicans*. VVC affects 75% of healthy women during childbearing age at least once, and an additional 5% to 10% of women suffer from recurrent VVC (RVVC), defined as 3 or more episodes of VVC per year (1). Predisposing factors for acute VVC include the use of high-estrogen oral contraception, hormone replacement therapy, frequent antibiotic use, and uncontrolled diabetes mellitus (2). Most RVVC cases, however, are idiopathic episodes that occur without recognizable predisposing factors (3). Symptoms of both VVC and RVVC include itching, burning, and redness of the vulva and vaginal mucosa accompanied by white vaginal discharge (3). Although antifungal therapies are typically effective in reducing fungal burden and symptoms, recurrence is common and requires maintenance regimens with antifungals (3). These recalcitrant symptoms along with rising costs to the health care system deleteriously affect the quality of life of women worldwide.

Previously, RVVC was thought to be attributable to defects in systemic and local cell-mediated immunity, similarly to other forms of candidiasis, in which protection against infection is T-cell dependent (reviewed in reference 4). However, numerous studies using a mouse model and cross-sectional clinical studies have shown that local or systemic adaptive immunity and associated cytokine/chemokines have no role in protection against vaginitis (5–10). Instead, innate immunity appears to play a role in both resistance and susceptibility to infection. Epithelial cells are postulated to provide some level of protection by inhibiting *C. albicans* growth, which limits colonization levels below the threshold required to elicit symptomatic infection (11–14). Conversely, symptomatic VVC is strongly associated with an acute inflammatory response characterized by polymorphonuclear leukocyte (PMN) recruitment to the vaginal lumen, which contributes to the symptoms but unfortunately does not clear the infection.

Recent studies using the experimental estrogen-dependent mouse model of *Candida* vaginitis revealed that the yeast-to-hypha switch and associated morphogenetic signaling pathways are critical contributors to PMN-mediated immunopathology (15). The response is initiated by *C. albicans-*epithelial cell interaction via the activity of pattern recognition receptors (PRRs) TLR4 and SIGNR1 that leads to vaginal epithelial cell-derived beta interleukin-1 (IL-1β) and S100A8 alarmin production. These mediators recruit PMNs to and activate them at the site of infection and serve to amplify the response (10, 15–18). Despite the strong inflammatory response to *C. albicans*, accumulating evidence clearly indicates that PMNs fail to reduce the fungal burden in the symptomatic disease state (15, 16, 18). Consequently, the lack of fungal clearance by PMNs confines the vaginal mucosa in a chronic inflammatory state. This has been shown for a variety of mouse strains (16, 19, 20) that can collectively be described as representative of chronic VVC-susceptible conditions (CVVC-S). Together, these findings imply a PMN dysfunction in the vaginal cavity due either to the inherent inability of the PMNs to adequately function or to an influence of the microenvironment on their function. In contrast, CD-1 mice cannot maintain *C. albicans* colonization following inoculation, presumably due to being inherently nonresponsive to exogenous estrogen, which is required to maintain colonization in CVVC-S mice (21, 22). However, the mechanism of fungal clearance has not been studied in detail. Nevertheless, the failure of estrogenized CD-1 mice to maintain *C. albicans* colonization following inoculation is suggestive of immune-based *C. albicans* clearance that can be defined as a chronic VVC-resistant condition (CVVC-R).

PMNs are among the primary components of host innate immune defense against *C. albicans* infection (23–25). The response primarily involves the recognition of Mac-1 (also termed α_M_β_2_, CD11b/CD18, and complement receptor 3) on PMNs with *C. albicans* pH-regulated antigen 1 protein (Pra1p), predominantly expressed on hyphae (26, 27). This Mac-1-mediated PMN activation has been shown to promote fungal killing by formation of neutrophil extracellular traps (NETs) (28). Although this activity appears to be absent during VVC, the requirement for hyphae in the immunopathogenic response (15) suggests that the components necessary for optimal fungal recognition by PMNs are present during infection. The objective of this study was to uncover the mechanisms of vaginal PMN dysfunction against *C. albicans* infection in VVC-susceptible mice.

## RESULTS

### PMN antifungal activity in CVVC-susceptible and -resistant mice.

**FIG 1:**
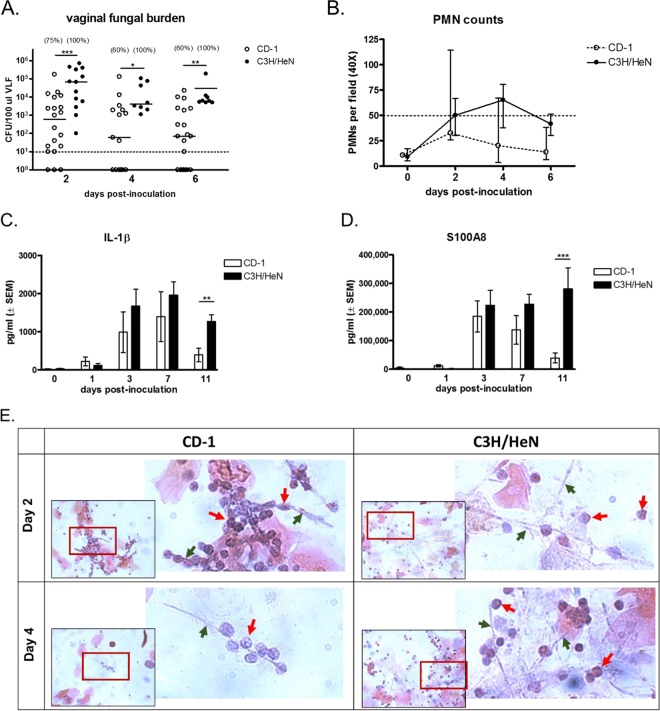

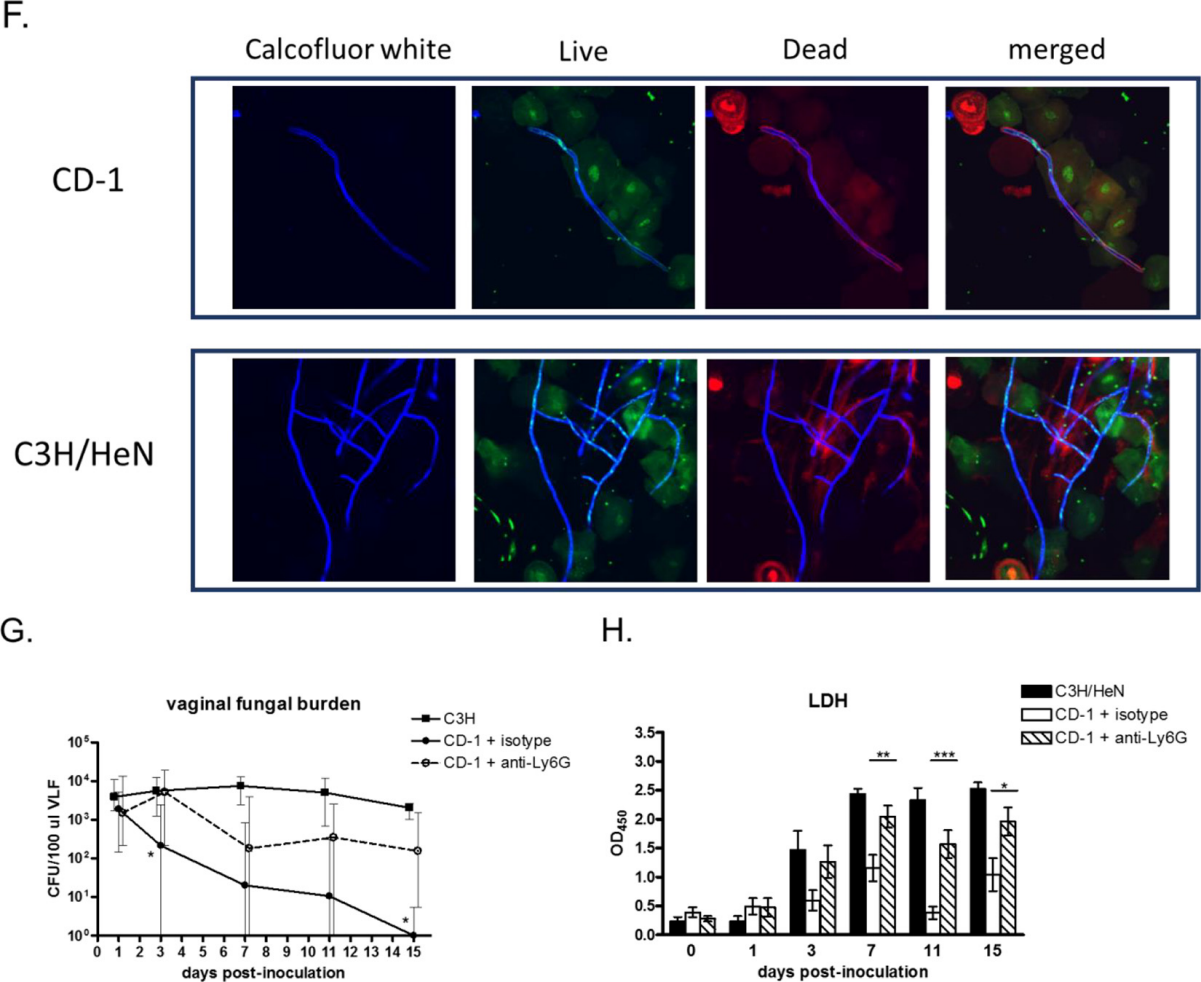
*C. albicans* colonization and PMN infiltration in the vaginal cavity of chronic VVC-susceptible and -resistant mice following inoculation. Estrogenized C3H/HeN (CVVC-S) and CD-1 (CVVC-R) mice were inoculated with 5 × 10^4^ *C. albicans* 3153A cells. (A) Vaginal fungal burden assessed longitudinally in vaginal lavage fluids (100 μl PBS/mouse) by quantitative plate count at indicated time points for a period of 6 days postinoculation. The dashed line denotes the infection threshold; vaginal fungal burden above the threshold of 10 CFU/100 μl lavage fluid was designated active colonization by *C. albicans*, and fungal burden below the threshold (<10 CFU) was considered negligible. Values in parentheses indicate percentages of mice colonized with *C. albicans* exhibiting >10 CFU. Horizontal bars represent medians. Data were analyzed using the Mann-Whitney *U* test at specific time points. (B) Vaginal lavage cellular infiltrates processed by the Pap smear technique examined by light microscopy. Numbers of PMNs were quantified in 5 different fields per sample and averaged. The dashed line denotes the arbitrary cutoff at which animals exhibiting 50 PMNs or above were defined as displaying the inflammatory condition (16). Data are expressed as medians and 25th/75th quartiles. CFU and PMN data are cumulative results from 4 independent experiments performed with 5 animals/group. (C and D) IL-1β (C) and S100A8 (D) in vaginal lavage fluids from estrogenized C3H/HeN and CD-1 mice quantified by ELISA over a period of 11 days postinoculation. Data were analyzed using the unpaired Student’s *t* test at specific time points. (E) Pap smear preparations of vaginal lavage fluids at 2 and 4 days postinoculation captured using a 40× objective (inset). The area indicated in red was expanded by digital zoom. Green arrows denote *C. albicans* hyphae, while red arrows denote PMNs. Images are representative of results from 4 independent experiments performed with 5 animals per group. (F) Cellular fractions of vaginal lavage fluids at 2 days postinoculation stained with calcofluor white (blue; *C. albicans*), SYTO 9 stain (green; live cells), and propidium iodide (red; dead cells) and observed by fluorescence microscopy. (G and H) Estrogenized CD-1 mice treated intraperitoneally with rat anti-mouse Ly6G antibodies to deplete PMNs or with rat IgG2A isotype control antibodies (100 μg/mouse) 1 day prior to intravaginal inoculation with *C. albicans* and every 3 days thereafter. Estrogenized C3H/HeN mice were inoculated and tested in parallel as positive controls. (G and H) Vaginal lavage fluids were assessed for fungal burden (median ± interquartile range) (G) and tissue damage as measured by lactate dehydrogenase (LDH) levels (H). CFU and LDH data represent cumulative results from 2 independent experiments performed with 10 animals/group. Data were analyzed by using the Mann-Whitney *U* test (G) or the unpaired Student’s *t* test (H) comparing anti-Ly6G-treated and isotype control-treated groups at specific time points. *, *P* < 0.05; **, *P* < 0.01; ***, *P* < 0.001; VLF, vaginal lavage fluid; PMN, polymorphonuclear leukocyte; SEM, standard error of the mean.

To confirm and further characterize disease progression under CVVC-S and CVVC-R conditions, groups of C3H/HeN (CVVC-S) and CD-1 (CVVC-R) mice were estrogenized and inoculated with *C. albicans* 3153A, and vaginal lavage fluids were evaluated longitudinally for fungal burden, PMN infiltration, and the presence of proinflammatory mediators (IL-1β and alarmin S100A8). CVVC-S C3H/HeN mice exhibited consistent *C. albicans* vaginal colonization at high levels in 100% of inoculated mice at all time points postinoculation. In contrast, vaginal fungal burden in CVVC-R CD-1 mice progressively declined throughout the observation period (*P* < 0.05 compared to levels in C3H/HeN mice at each time point), with 40% of CD-1 mice showing clearance by 4 days postinoculation (Fig. 1A). In line with previous observations (16), C3H/HeN mice displayed increasing levels of PMN infiltration beginning 48 h postinoculation and largely continuing throughout the observation period. In contrast, while CD-1 mice showed a similar increase in PMN infiltration through 48 h postinoculation, a steady decline was observed thereafter that was consistent with the reduction in fungal burden. However, this decline did not reach significance at day 4 or 6 compared to the results seen with CVVC-S mice (Fig. 1B). Consistent with the PMN migration, dramatic increases in levels of IL-1β (Fig. 1C) and S100A8 (Fig. 1D) were observed through 7 days postinoculation in both strains of mice. However, while the elevated levels of IL-1β and S100A8 were sustained throughout the remainder of the observation period in C3H/HeN mice, the levels of each were significantly decreased in CD-1 mice compared to C3H/HeN mice at 3 days postinoculation onward to 11 days (*P* < 0.008 [IL-1β] and *P* < 0.0008 [S100A8]).

In order to determine whether the reduced vaginal fungal burden was associated with the presence of PMNs and their antifungal capacity, cellular fractions of vaginal lavage fluid from C3H/HeN and CD-1 mice on days 2 and 4 postinoculation were examined by microscopy. First, Pap smear (Papanicolaou technique) preparations of cells harvested from the vagina of each mouse strain were visually evaluated for the interaction of PMNs with *C. albicans*. In samples from inoculated C3H/HeN mice, a mix of hypha-associated and nonassociated PMNs was observed in microscopic fields on days 2 and 4 postinoculation. In contrast, PMNs in CD-1 mice were intimately interacting with *C. albicans* hyphae on day 2 and, if present, also on day 4 (Fig. 1E). Next, the viability of *C. albicans* in vaginal lavage fluid from inoculated C3H/HeN and CD-1 mice on day 2 postinoculation was determined by cell viability staining and visualized by confocal microscopy. Images obtained from inoculated CD-1 mice showed the presence of nonviable damaged hyphae, which stain positively with propidium iodide (red) (Fig. 1F). Similar results were observed in CD-1 mice on day 4 postinoculation (data not shown). In contrast, hyphae from C3H/HeN mice stained positively only with SYTO 9 (green), indicating intact cell membranes and lack of damage.

To determine whether the presence of vaginal PMNs and inflammatory mediators in CVVC-R CD-1 mice was involved in the reduction of fungal burden, estrogenized CD-1 mice were administered rat anti-mouse Ly6G antibody to deplete PMNs prior to inoculation. As previously reported (15), PMN depletion was confirmed by microscopic evaluation in vaginal lavage fluid at each time point postinoculation compared to the results seen with the mice that received isotype control antibody. Vaginal fungal burden and the activity of lactate dehydrogenase (LDH) (a known marker of tissue damage) were assessed in vaginal lavage fluid from antibody-treated CD-1 mice compared to CVVC-S C3H/HeN mice. Similarly to the results shown in Fig. 1A, isotype-treated CD-1 mice failed to maintain fungal colonization, exhibiting a steady decline in vaginal fungal burden throughout the observation period (Fig. 1G). In contrast, anti-Ly6G-treated CD-1 mice maintained colonization similar to that seen with C3H/HeN mice, exhibiting increased vaginal fungal burden compared to isotype-treated CD-1 mice on day 3 and 15 postinoculation (*P* < 0.05). Furthermore, anti-Ly6G-treated CD-1 mice showed a sharp increase in LDH activity similar to that seen with C3H/HeN mice starting at 3 days postinoculation that continued throughout the observation period, whereas LDH levels in isotype-treated CD-1 mice were significantly reduced compared to those in anti-Ly6G-treated mice on days 7, 11, and 15 postinoculation (*P* < 0.05) (Fig. 1H).

### *In vivo* effect of elicited vaginal PMNs during vaginitis.

**FIG 2:**
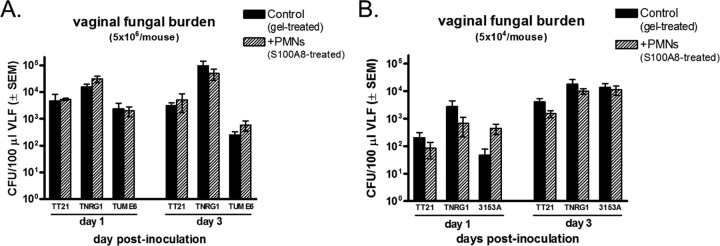
Vaginal fungal burden in inoculated mice under preinduced inflammatory conditions in the vagina. Overnight culture supernatants of S100A8-producing *C. albicans* were semisolidified with carboxymethylcellulose (3%) to obtain a consistency of vaginal gel. The gel preparations of S100A8 or vehicle alone were administered intravaginally to estrogenized uninoculated mice daily for 3 days in a volume of 20 μl per mouse using a microdispenser. The induction of PMN recruitment in the vagina was confirmed by identifying PMNs in vaginal lavage fluids (preinoculation) using the Pap smear technique. Mice were inoculated with 5 × 10^6^ *C. albicans* TNRG1 cells (yeast locked) or TUME6 (hypha-locked) or TT21 (wild-type strain) cells (A) or with 5 × 10^4^ *C. albicans* TNRG1, TT21, or 3153A (standard laboratory strain; standard inoculum) (B). Vaginal lavage samples were collected on days 1 and 3 postinoculation, and vaginal fungal burden was assessed by quantitative plate count. Data were analyzed using an unpaired Student’s *t* test at each time point. The figure represents cumulative results from 2 independent experiments performed with 5 animals per group. VLF, vaginal lavage fluid; PMN, polymorphonuclear leukocyte; SEM, standard error of the mean.

Previous studies showed that intravaginal administration of recombinant S100A8 alarmin was sufficient to induce PMN migration into the vaginal cavity in the absence of inoculation (18). This strategy was used to test whether the presence of preelicited PMNs in the vagina at the time of inoculation could reduce *C. albicans* fungal burden *in vivo*. For this, estrogenized C3H/HeN mice were pretreated with recombinant S100A8, while control mice received vehicle alone. Upon confirmation of vaginal PMN recruitment, mice were inoculated with *C. albicans* strain TT21 (wild type), strain TNRG1 (yeast locked), or strain TUME6 (hypha locked), and vaginal fungal burden was evaluated on days 1 and 3 postinoculation. Results showed that the *C. albicans* vaginal fungal burden was not affected by preelicited PMN recruitment for any of the fungal strains (Fig. 2A). The same approach was employed using a suboptimal inoculum (2 logs lower) with *C. albicans* TT21 and TNRG1 in parallel with 3153A (standard laboratory strain). Again, comparable levels of vaginal fungal burden were observed between mice with preelicited PMNs and control mice for each fungal strain (Fig. 2B).

### Killing capacity of elicited peritoneal and vaginal PMNs under vaginal simulation conditions *in vitro*.

**FIG 3:**
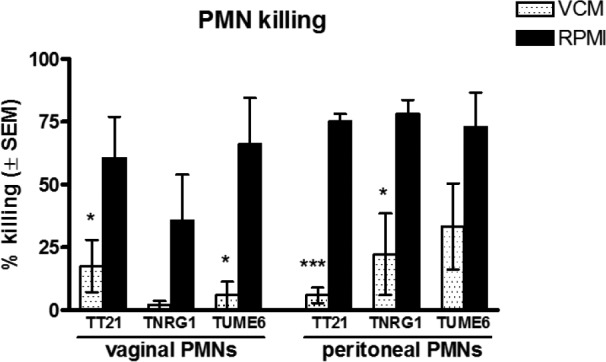
*In vitro* killing activity of peritoneally and vaginally elicited PMNs against *C. albicans* in standard or vaginal conditioned medium. Elicited peritoneal PMNs were harvested from peritoneal exudate cells of casein-treated C3H/HeN mice. Elicited vaginal PMNs were obtained by enriching Gr-1/Ly6G^+^ leukocytes in vaginal lavage fluid from S100A8-treated mice by the magnetically activated cell sorting technique. Vaginal conditioned medium (VCM) was prepared by conducting vaginal lavage in estrogenized C3H/HeN mice using 100 μl of RPMI 1640 per mouse as a lavage vehicle. Elicited peritoneal and vaginal PMNs (5 × 10^5^) were examined for *in vitro* killing activity by coculturing with 5 × 10^4^ *C. albicans* TNRG1 (yeast-locked), TUME6 (hypha-locked), or TT21 (wild-type) cells in 100 μl of pooled VCM or RPMI medium for 3 h at 37°C with 5% CO_2_. Cells of each *C. albicans* strain were cultured alone in VCM or RPMI medium in parallel as controls. Viable *C. albicans* cells were enumerated by quantitative plate count. Data were analyzed using an unpaired Student’s *t* test. The figure represents cumulative results from 3 independent experiments. *, *P* < 0.05; ***, *P* < 0.001; VCM, vaginal conditioned medium; PMN, polymorphonuclear leukocyte; SEM, standard error of the mean.

In order to explore possible underlying mechanisms for the lack of fungal clearance by PMNs in the vagina, we modified an established *in vitro* PMN killing assay to assess activity under vaginal simulation conditions that would include biologically relevant secretory factors present in the vagina. For this, vaginal lavage of estrogenized C3H/HeN CVVC-S mice using RPMI medium (standard medium in the killing assay) was used to obtain vaginal conditioned medium (VCM). The VCM was incorporated into the evaluation of elicited peritoneal and vaginal PMNs from estrogenized mice, for analysis of killing activity against *C. albicans* wild-type, yeast-locked, or hypha-locked strains compared to standard RPMI medium. Results showed that elicited peritoneal and vaginal PMNs had equivalent levels of killing capacity against yeast and hyphal forms of *C. albicans* in standard media (Fig. 3). In contrast, both elicited peritoneal and vaginal PMNs cultured in VCM demonstrated significantly reduced antifungal activity against both *C. albicans* yeast and hyphae compared to PMNs cultured in the standard media (*P* < 0.05 [vaginal PMNs with TT21 and TUME6] and *P* < 0.05 [peritoneal PMNs with TT21 and TNRG1]) (Fig. 3). Importantly, to rule out the possibility that the inhibitory activity of VCM was due to depletion/dilution (via the vaginal lavage) of medium components that are important for the *in vitro* killing assay, inhibitory VCM was supplemented with concentrated RPMI medium and evaluated in the assay. Similarly, RPMI medium was subjected to protease/heat treatment prior to use in the assay as another control. Results showed that VCM-dependent inhibition of *C. albicans* killing was unaffected by RPMI medium supplementation and that protease/heat treatment of RPMI medium had no effect on normal PMN killing of *C. albicans* (data not shown).

### Biological properties of VCM and estrogen for the PMN functional defect against *C. albicans in vitro*.

**FIG 4:**
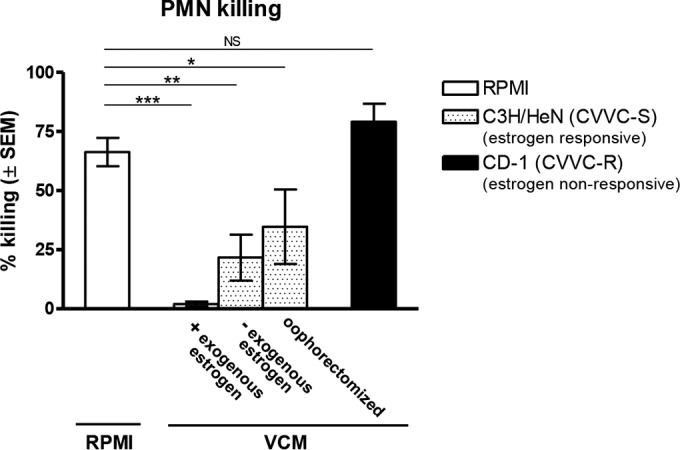
Limiting requirements in the VCM for the inhibition of PMN killing activity against *C. albicans*. VCM represented pooled vaginal lavage fluids from estrogenized, untreated, or oophorectomized C3H/HeN mice (CVVC-S, estrogen responsive) and untreated CD-1 mice (CVVC-R, estrogen nonresponsive), and 100 μl of RPMI 1640 per mouse was used as a lavage vehicle. Elicited peritoneal PMNs (5 × 10^5^) were cocultured with 5 × 10^4^ *C. albicans* 3153A cells in 100 μl of pooled VCM or RPMI medium for 3 h at 37°C with 5% CO_2_ and examined for *in vitro* killing activity. *C. albicans* cells were cultured alone in each medium and tested in parallel as controls. Viable *C. albicans* cells were enumerated by quantitative plate count. Data were analyzed using an unpaired Student’s *t* test. The figure represents cumulative results from 3 independent experiments. *, *P* < 0.05; **, *P* < 0.01; NS, not significant; VCM, vaginal conditioned medium; PMN, polymorphonuclear leukocyte; SEM, standard error of the mean.

On the basis of the observation that VCM inhibited killing of *C. albicans* by PMNs from different anatomical sites, it was important to determine the limiting factors of the vaginal environment for the inhibitory activity. Accordingly, VCM was obtained from naive C3H/HeN (CVVC-S) mice with or without exogenous estrogen treatment, as well as from oophorectomized C3H/HeN mice, and tested in the PMN killing assay using elicited peritoneal PMNs. VCM was also obtained from naive CD-1 (CVVC-R) mice with or without exogenous estrogen treatment and tested in parallel. As shown in Fig. 4, the antifungal capacity of PMNs cocultured in VCM from C3H/HeN mice was stunted to a degree equal to that seen with standard media independently of the presence of exogenous or endogenous estrogen (*P* = 0.0001, *P* = 0.001, and *P* = 0.036 for the estrogenized, nonestrogenized, and oophorectomized groups, respectively). In contrast, PMNs cocultured in VCM from naive CD-1 mice exhibited antifungal activity similar to that seen with PMNs in standard media (*P* = 0.26). Similar results were observed using VCM from CD-1 mice given exogenous estrogen (data not shown).

### Presence of putative inhibitory factor(s) in VCM under the CVVC-susceptible conditions.

To further investigate the properties of PMN dysfunction observed under the vaginal simulation conditions, VCM from naive C3H/HeN mice was subjected to protease treatment followed by heat inactivation and was then evaluated for effects on PMN antifungal activity. Results showed that killing of *C. albicans* by PMNs could be restored using protease/heat-treated VCM (67% ± 15% fungal killing in protease/heat-treated VCM versus 24% ± 11% in untreated VCM; *P* = 0.026).

### Evidence that the putative inhibitory factor in VCM is a ligand for the PMN receptor Mac-1.

**FIG 5:**
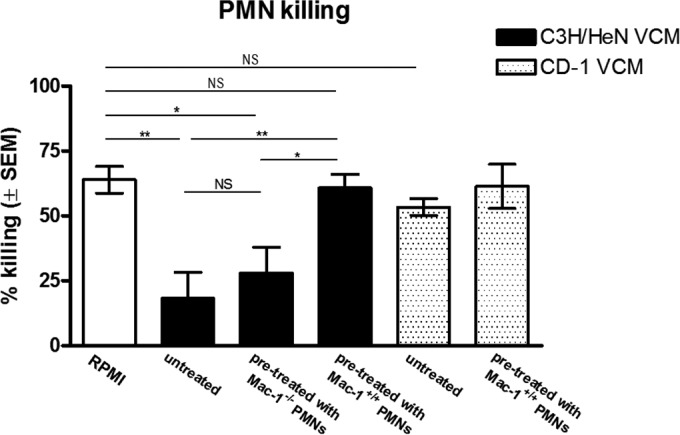
Role of PMN receptor Mac-1 as a ligand for VCM inhibitory factor. VCM represented pooled vaginal lavage fluids from untreated C3H/HeN (CVVC-S) or CD-1 (CVVC-R) mice using 100 μl of RPMI 1640 per mouse as a lavage vehicle. VCM was preincubated with 1 × 10^6^ Mac-1^−/−^- or Mac-1^+/+^-elicited peritoneal PMNs collected from Mac-1 KO or wild-type C57BL/6 mice, respectively, for 1 h at 37°C. PMNs were removed from VCM by centrifugation following incubation. The recovered VCM (pretreated), untreated VCM, or RPMI medium was tested in the PMN killing assay using freshly isolated elicited peritoneal wild-type PMNs (5 × 10^5^) and challenged with *C. albicans* (5 × 10^4^) in a volume of 100 μl for 3 h at 37°C with 5% CO_2_. *C. albicans* cells were cultured alone in each medium and tested in parallel as controls. Viable *C. albicans* cells were enumerated by quantitative plate count. Data were analyzed using an unpaired Student’s *t* test. The figure represents cumulative results from 4 independent experiments. *, *P* < 0.05; **, *P* < 0.01; NS, not significant; VCM, vaginal conditioned medium; PMN, polymorphonuclear leukocyte; SEM, standard error of the mean.

**FIG 6:**
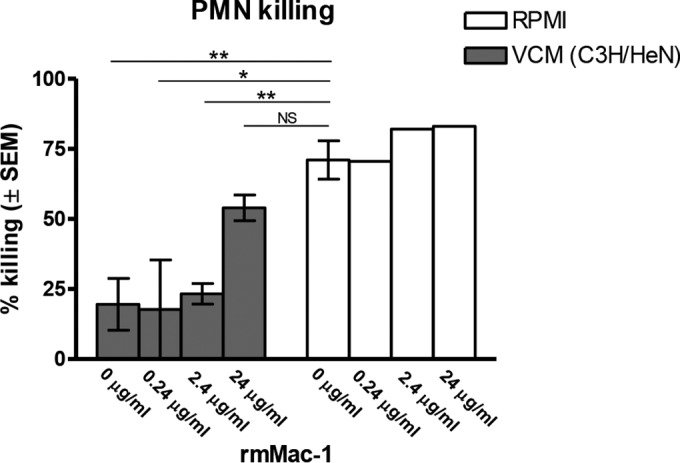
Effect of recombinant Mac-1 as a competitive inhibitor to rescue PMN killing of *C. albicans* in VCM. VCM represented pooled vaginal lavage fluids from untreated C3H/HeN (CVVC-S) mice using 100 μl of RPMI 1640 per mouse as a lavage vehicle. VCM or RPMI medium was pretreated with recombinant mouse Mac-1 (rmMac-1) at the concentrations indicated for 1 h at 37°C. The pretreated VCM or RPMI medium was tested in the PMN killing assay using elicited peritoneal PMNs (5 × 10^5^) and challenged with *C. albicans* (5 × 10^4^) in a volume of 100 μl for 3 h at 37°C with 5% CO_2_. *C. albicans* cells were cultured alone in each medium and tested in parallel as controls. Viable *C. albicans* cells were enumerated by quantitative plate count. Data were analyzed using an unpaired Student’s *t* test. The figure represents cumulative results from 3 independent experiments. *, *P* < 0.05; **, *P* < 0.001; NS, not significant; VCM, vaginal conditioned medium; PMN, polymorphonuclear leukocyte; SEM, standard error of the mean.

On the basis of data suggesting that a putative inhibitory factor(s) is present in VCM, we next sought to determine a potential mechanism by which PMN antifungal activity might become inhibited under the CVVC-susceptible conditions. The PMN receptor Mac-1 has been reported to represent a critical ligand for fungal recognition via Pra1p (pH-regulated antigen 1 protein) expression on *C. albicans* hyphae (27) that initiates PMN-mediated killing (29). We hypothesized that an inhibitory factor present in the CVVC-susceptible vaginal environment blocks the interaction of Mac-1 with a fungal cell surface molecule, potentially Pra1p. To begin to test this hypothesis, we investigated whether the putative inhibitory factor in VCM could be depleted by Mac-1^+/+^ PMNs, thereby allowing PMN killing. For this, inhibitory VCM was pretreated with Mac-1^−/−^ or Mac-1^+/+^ PMNs collected from Mac-1 knockout (KO) or wild-type mice, respectively, and evaluated for PMN antifungal activity. Following pretreatment with Mac-1^−/−^ PMNs, VCM retained its inhibitory effect at a level similar to that seen with untreated VCM (*P* = 0.53). In contrast, the antifungal activity of PMNs cultured in VCM pretreated with Mac-1^+/+^ PMNs showed enhanced killing compared to PMNs in untreated VCM (*P* = 0.0097) that was representative of the levels observed for PMNs cultured in standard media (Fig. 5). Importantly, pretreatment of CD-1 (noninhibitory) VCM with Mac-1^+/+^ PMNs had no effect on PMN killing activity; the activity was similar to that seen with untreated CD-1 VCM and standard media (*P* = 0.3 and *P* = 0.8, respectively). A second approach was also used whereby inhibitory VCM was pretreated with recombinant soluble mouse Mac-1 at various concentrations to neutralize putative Mac-1 blocking ligands and then evaluated in the PMN killing assay. Results showed that PMN killing of *C. albicans* was restored in a dose-dependent manner following pretreatment of VCM with recombinant Mac-1; pretreatment with 24 µg/ml had activity similar to that of the standard medium (*P* = 0.013), whereas pretreatment with 2.4 µg/ml or less had reduced activity compared to the standard medium (*P* < 0.05) (Fig. 6).

**FIG 7:**
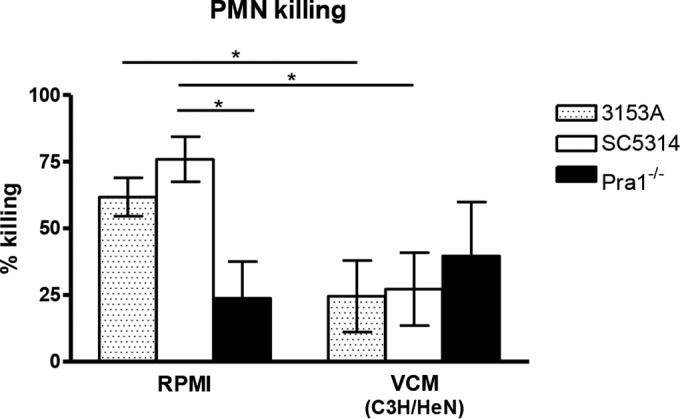
Confirmation of *C. albicans* Pra1p as the major ligand of Mac-1 required for PMN-mediated antifungal activity. VCM represented pooled vaginal lavage fluids from untreated C3H/HeN (CVVC-S) mice using 100 μl of RPMI 1640 per mouse as a lavage vehicle. Elicited peritoneal PMNs (5 × 10^5^) were cocultured with 5 × 10^4^ *C. albicans* Pra1^−/−^, wild-type SC5314, or the 3153A (assay control) strain in 100 μl of pooled VCM or RPMI medium for 3 h at 37°C with 5% CO_2_ and examined for *in vitro* killing activity. *C. albicans* cells were cultured alone in each medium and tested in parallel as controls. Viable *C. albicans* cells were enumerated by quantitative plate count. Data were analyzed using an unpaired Student’s *t* test. The figure represents cumulative results from three independent experiments. *, *P* < 0.05; VCM, vaginal conditioned medium; PMN, polymorphonuclear leukocyte; SEM, standard error of the mean.

To further confirm the interaction of Mac-1 and Pra1p in the induction of PMN antifungal activity in the vaginal environment, we used a *PRA1-*deficient (Pra1^−/−^) *C. albicans* strain in the *in vitro* PMN killing assay and for *in vivo* inoculation in mice. In a first series of studies, peritoneal PMNs were cocultured with *C. albicans* Pra1^−/−^, wild-type (SC5314), or 3153A (assay control) strains in standard media or inhibitory VCM from C3H/HeN mice and evaluated for PMN killing activity. As expected, PMNs had equivalent high levels of killing capacity against wild-type and 3153A *C. albicans* in standard media, while killing of Pra1^−/−^ *C. albicans* was significantly reduced (*P* = 0.035 compared to 3153A; *P* = 0.018 compared to SC5314). In contrast, levels of PMN killing of 3153A, SC5314, and Pra1^−/−^ *C. albicans* were all similarly reduced in VCM (Fig. 7). A second series of studies evaluated the effect of the Pra1 deletion on the fate of *C. albicans* burden in estrogenized CD-1 (CVVC-R) mice over a 10-day period. Results showed that while mice inoculated with the wild-type strain had a steady decline (~2 logs) of fungal burden over the 10-day period (similar to the results shown in Fig. 1A), mice inoculated with the Pra1^−/−^ mutant strain maintained a steady level of fungal burden throughout, albeit ~1 to 2 logs lower than that observed with the wild-type strain (data not shown).

### Heparan sulfate as a putative Mac-1 competitive inhibitor in VCM.

**FIG 8:**
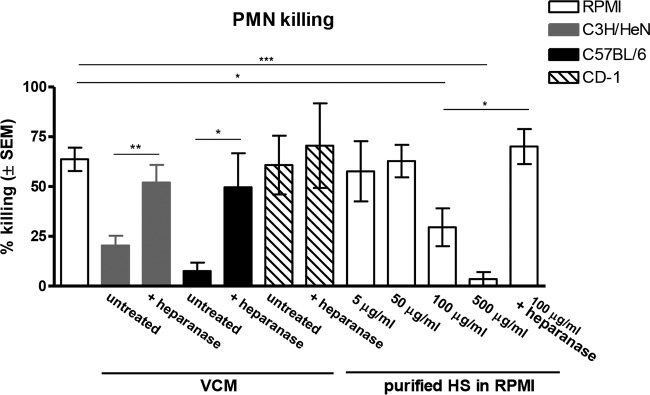
Heparan sulfate as the putative inhibitory factor in VCM affecting PMN function against *C. albicans*. VCM represented pooled vaginal lavage fluids from untreated C3H/HeN (CVVC-S), C57BL/6 (CVVC-S), or CD-1 (CVVC-R) mice using 100 μl of RPMI 1640 per mouse as a lavage vehicle. VCM was pretreated with heparinase III (5 U/ml) for 1 h at 37°C and subjected to the PMN killing assay by coculturing elicited peritoneal PMNs (5 × 10^5^) and *C. albicans* (5 × 10^4^) for 3 h at 37°C with 5% CO_2_. Purified heparan sulfate (HS) was diluted in RPMI medium to the concentrations indicated. HS-containing medium was used in the 3-h PMN killing assay. A control included pretreated HS media at 100 μg/ml with heparanase. *C. albicans* cells were cultured alone in each medium as controls. Viable *C. albicans* cells were enumerated by quantitative plate count. Data were analyzed using an unpaired Student’s *t* test. The figure represents cumulative results from three independent experiments. *, *P* < 0.05; **, *P* < 0.01; ***, *P* < 0.001; VCM, vaginal conditioned medium; PMN, polymorphonuclear leukocyte; SEM, standard error of the mean.

Mac-1 has the capacity to bind multiple ligands and regulate various leukocytic functions (30–32). There are reports showing that heparan sulfate (HS) is one such ligand for Mac-1 that could compete for binding of Mac-1 ligands necessary for PMN activation (33, 34). In addition, HS is expressed in mouse vaginal epithelium and human vaginal epithelial cell cultures (35–37). To test the potential of HS as a competitive inhibitor for the Mac-1/Pra1p-associated PMN activity against *C. albicans*, inhibitory VCM from naive C3H/HeN mice was treated with heparanase (heparinase III, specific for HS) and then evaluated in the PMN killing assay. Results showed that treatment of VCM with heparanase increased PMN killing of *C. albicans* (*P* = 0.0095 compared with untreated VCM) to levels comparable to those observed for standard media (Fig. 8). Treatment of VCM with heparinase I (inactive for HS) had no effect on killing inhibition (data not shown). Similar results were observed using VCM from C57BL/6 mice (*P* = 0.027 compared to untreated VCM) consistent with their estrogen-dependent susceptibility to chronic VVC (16, 19, 20). In contrast, heparanase treatment of CD-1 (noninhibitory) VCM had no effect on PMN killing activity (*P* = 0.72 compared to untreated VCM). To further confirm the inhibitory effect of HS on PMN antifungal activity, standard culture medium was spiked with purified HS at various concentrations and tested in the PMN killing assay in comparison to inhibitory (C3H/HeN) and noninhibitory (CD-1) VCM. Results showed inhibition of PMN killing by HS in a dose-dependent manner compared to the vehicle control (RPMI medium) results (*P* = 0.014 at 100 µg/ml; *P* = 0.0002 at 500 µg/ml). Similar to observations for VCM, HS-mediated (100 µg/ml) inhibition of PMN killing was abrogated by heparanase treatment (*P* = 0.033) (Fig. 8).

## DISCUSSION

Despite the recruitment of PMNs into the vaginal cavity in response to *C. albicans* and strong evidence for their role in the hallmark immunopathology and ensuing symptomatology in those susceptible to VVC (16, 38), there are no solid explanations for the lack of effects on fungal burden. This led us to hypothesize that PMNs fail to exert antifungal activity in the vagina due to the presence of a *Candida*-tolerogenic vaginal microenvironment. Here, through the use of an established animal model incorporating CVVC-S C3H/HeN (nonclearing) mice (16, 19, 20) and uniquely CVVC-R CD-1 (clearing) mice (21, 22), we identified the putative mechanism of vaginal PMN dysfunction during VVC.

We have also confirmed that CVVC-R CD-1 mice are able to clear *C. albicans* infection following vaginal PMN recruitment, which was associated with close physical interaction of PMNs with *Candida* and reduced fungal burden compared to CVVC-S C3H/HeN mice. The CD-1 mice, although outbred and potentially likely to give variable responses with respect to experimental approaches, were very consistent overall in VVC parameters and thus served as a reproducible control strain for the resistant/clearing phenotype. In line with previous reports (15, 16), the VVC-associated inflammatory mediators that were present in colonized mice reached a plateau level as early as 3 days postinoculation, suggesting that acute inflammatory responses are triggered under both CVVC-R and CVVC-S conditions. However, distinct differences in the levels of inflammatory mediators were evident at later times postinoculation; levels in CD-1 mice declined to near the baseline levels by 11 days in a manner concomitant with similar reductions in PMN migration and fungal burden, whereas all of the VVC parameters were sustained in C3H/HeN mice. A direct role for PMNs in the reduced fungal burden in CD-1 mice was confirmed by PMN depletion. Furthermore, PMN-mediated fungal clearance observed in CD-1 mice resulted in minimal tissue damage (as measured by LDH levels) whereas significantly higher levels were observed in C3H/HeN mice and CD-1 mice depleted of PMNs. Taking these data together, the evidence strongly supports the hypothesis that susceptibility to VVC is due to a lack of fungal clearance by PMNs and to chronic inflammation in the vaginal cavity. The CVVC-R and CVVC-S strains of mice are also representative of clinically relevant conditions of women resistant or susceptible to VVC/RVVC prior to any antifungal intervention.

For the CVVC-S C3H/HeN mice, the lack of reduction in vaginal fungal burden when PMNs were present and activated prior to inoculation, even under conditions of suboptimal inocula or different morphological forms of *C. albicans*, suggested that the PMNs were defective in antifungal activity in the vaginal microenvironment. However, vaginal PMNs functioned equivalently to peritoneal PMNs outside the vaginal environment, displaying significant antifungal activity against all morphological forms of *C. albicans*. This suggested that vaginal PMNs are not inherently dysfunctional but that factors in the vaginal microenvironment are inhibiting their antifungal activity. This was confirmed by the ability of vaginal conditioned medium (VCM) from two strains of CVVC-S mice (C3H/HeN and C57BL/6) to inhibit PMN killing of *C. albicans*. Further support came from the fact that PMNs functioned normally in VCM from CVVC-R CD-1 mice. This suggests that the mechanism of clearance in CD-1 mice is directly related to the ability of PMNs to exert adequate antifungal activity and that such activity can occur *in vivo* in the vaginal environment.

The inhibitory activity of VCM from CVVC-S mice was not dependent on the presence of endogenous or exogenous estrogen but was dependent on estrogen responsiveness. C3H/HeN mice are highly estrogen responsive, as are all other CVVC-S mouse strains previously evaluated (16, 19). CD-1 mice, on the other hand, have significantly reduced estrogen responsiveness (21, 39). Although estrogen hyposensitivity in CD-1 females was not tested in this study, reports by Calderon et al. demonstrated that resistance to sustained colonization in CD-1 mice is independent of the *C. albicans* strain, the commercial mouse supplier, and the major histocompatibility complex (MHC) haplotype (22). Thus, we propose that estrogen responsiveness and its impact on the vaginal microenvironment are key underlying factors for PMN dysfunction under CVVC-S conditions and are manifested in vaginal secretions. The fact that the inhibitory factor was sensitive to protease and heat treatment indicated that a soluble protein or protein-associated moiety present in vaginal secretions was interfering with PMN function.

Based on the key interaction between Mac-1 and *C. albicans* Pra1p for PMN killing activity (27, 29), we hypothesized that the protein or protein-associated moiety in VCM was interfering with the Pra-1p/Mac-1 interaction. Indeed, the rescue of PMN activity following absorption of VCM with Mac-1^+/+^ (but not Mac-1^−/−^) PMNs suggested that the inhibitory factor in VCM was a Mac-1 ligand. This was further confirmed using recombinant soluble Mac-1 in a similar design. In addition, use of Pra1^−/−^ *C. albicans* allowed us to confirm the requirement for Mac-1/Pra1p interaction in PMN-mediated antifungal activity. This was shown most strongly in the *in vitro* PMN killing assay. For the *in vivo* studies, the SC5314 parent strain is a notoriously poor colonizer of mucosal tissues (40) and the Pra1^−/−^ mutant was even worse in the VVC model. This may have been due to the fact that Pra1p also acts as a major adhesion molecule in concert with other *C. albicans* proteins (27, 41). Yet we were able to show a low sustained vaginal fungal burden in CD-1 mice inoculated with the Pra1^−/−^ strain, whereas the SC5314 parent strain showed a progressive decline similar to that shown by the laboratory strain (3153A), suggestive of the escape from killing by PMNs in the absence of Mac-1/Pra1p interaction. This observation is further supported by evidence that the Mac-1/Pra1p interaction occurs primarily with *C. albicans* hyphae (27) and that the morphological transition to hyphae is required to induce PMN migration in the mouse model (15).

We subsequently hypothesized that the proteoglycan HS in VCM was the putative inhibitory factor, on the basis of the fact that HS is a reported ligand for Mac-1 and of its presence in various tissues, including vaginal epithelium (33–37). Data showing a restoration of PMN antifungal activity following treatment of VCM with heparanase strongly supported this hypothesis. This was confirmed by the dose-dependent inhibition of killing activity by purified HS in standard culture medium. It is unclear how the Mac-1/Pra-1 interaction mediates fungal killing, but it may be related to formation by PMNs of neutrophil extracellular traps (NETs), which are complexes composed of DNA laced with antimicrobial proteins that are triggered to be released upon recognition of pathogens. NET formation is a potent antifungal defense mechanism *in vitro* and *in vivo* in other models of candidiasis (42–44). It will therefore be interesting to examine NETs in CVVC-S and CVVC-R mice.

HS is a member of the glycosaminoglycan family of polysaccharides ubiquitously expressed in a proteoglycan form on cell surfaces and throughout the extracellular matrix in all mammalian tissues (45). HS proteoglycans consist of a protein core attached with one or more HS chains, and a wide range of modifications are possible, resulting in a remarkable structural diversity due to variations in chain length (46). HS proteoglycans are known to be involved in diverse biological processes, including wound healing, angiogenesis, and inflammation, as well as in binding to both host and microbial components, although no evidence of interaction with fungi has been reported (46). In epithelial tissues, stratified squamous epithelial cells (representing an estrogen-responsive cell type in the vagina) express much higher levels of HS proteoglycans on the entire cell surface whereas expression is restricted in columnar epithelial cells (36). Interestingly, tissue-specific structural polymorphism in proteoglycan expression has been previously shown. Reports by Sanderson and Bernfield demonstrated that proteoglycans on vaginal epithelial cells had smaller HS chains than those expressed at other anatomical sites with identical core proteins such as the gastrointestinal tract, leading to production of divergent molecular structures (36). This diversity in HS expression could explain the dichotomous results seen with VCM from CVVC-R CD-1 and CVVC-S C3H/HeN mice, potentially reflecting differences in relative levels of binding to Mac-1. Other explanations include the absence of HS or lower concentrations of HS in CD-1 mice. The lack of effective methods to accurately quantify HS in biological fluids is currently limiting the ability to formally address this issue.

Although our results strongly support the concept of HS as a competitive inhibitor of PMN function in the vaginal environment, there is also the possibility that *C. albicans* biofilm formation has a negative influence on PMN antifungal capacity. Because *C. albicans* has been shown to form biofilms on the vaginal mucosa in mice (47), PMNs may exert reduced killing activity, as has been shown *in vitro* and with other *in vivo* candidiasis models (48–50). However, a biofilm-deficient *C. albicans* mutant (*bcr1*^*−/−*^) colonized as well as wild-type *C. albicans* during vaginal infection independently of PMN infiltration (15). This suggests that PMN dysfunction cannot be explained by vaginal biofilm formation.

A recent publication described a similar PMN inhibitory factor in lavage fluid of CVVC-R CD-1 mice during/following infection with *C. albicans* (51). Thus, there may be inducible PMN inhibitory factors present during the natural history of a vaginal infection. Interesting, while we have data supporting these findings in infected CD-1 mice, the killing activity could not be rescued or increased following treatment of the vaginal lavage with heparanase (data not shown). Hence, the inducible inhibitory factor appears to function via a mechanism that is independent of HS.

On the basis of the current findings, HS may represent a novel marker capable of exploitation for therapeutic strategies. Accordingly, future studies will include HS neutralization by intravaginal administration of soluble recombinant Mac-1 or heparanase to CVVC-S mice during infection to improve PMN antifungal activity. We recognize, however, that caution must be taken with this approach, as HS is multifunctional and neutralization may interfere with other protective or nonprotective processes in the vaginal environment (i.e., may promote or inhibit viral binding and entry into host cells) (52, 53). The reverse experiments, involving intravaginal administration of HS in CVVC-R CD-1 mice to inhibit PMN function, will also shed light on HS as a therapeutic target.

In addition to mechanistic and therapeutic studies in the mouse model, it will be important to test the hypotheses in a clinical setting. We have preliminary evidence that, similar to the observation for mouse PMNs, human PMNs have reduced antifungal activity in mouse-inhibitory VCM (data not shown). Moreover, based on the similarity between mouse and humans in the acute inflammatory response and associated symptomatology (16, 38), we predict that VCM from women susceptible to VVC/RVVC will exhibit a similar inhibitory effect on PMN function by the same mechanism: HS-mediated competitive inhibition of Mac-1. Indeed, results from a small clinical pilot study revealed that killing by human and mouse PMNs in the presence of VCM preparations from women with a history of VVC could be significantly enhanced following treatment of the VCM with heparanase (P. L. Fidel, unpublished data). In contrast, we predict that women with no history of VVC (i.e., resistant women) have no/low levels of HS, have different forms of HS, or do not trigger PMN migration due to low epithelial cell sensitivity to *C. albicans*.

In conclusion, we have demonstrated that HS in vaginal secretions is linked to PMN dysfunction in the vaginal microenvironment as a putative competitive Mac-1 ligand that interferes with the ability of PMNs to optimally interact with *Candida* for killing. Continued work in the animal model together with clinical studies will be invaluable to confirm the mechanism of PMN dysfunction and to explore the potential of immunotherapeutic targeting of Pra-1p/Mac-1/HS interactions.

## MATERIALS AND METHODS

### Mice.

Female C3H/HeN (CVVC-susceptible) mice, oophorectomized C3H/HeN mice, and CD-1 (CVVC-resistant) mice, 5 to 7 weeks of age, were purchased from Charles River Laboratories, Inc. (Wilmington, MA). Female Mac-1 knockout (KO) mice, 5 to 7 weeks of age, and age-matched C57BL/6 (CVVC-S) (wild-type) mice were purchased from Jackson Laboratories (Bar Harbor, ME). All animals were housed and handled according to institutional recommended guidelines. All animal protocols were reviewed and approved by the Institutional Animal Care and Use Committee (IACUC) of the LSU Health Sciences Center, New Orleans, LA.

### *C. albicans* strains.

*C. albicans* 3153A (the National Collection of Pathogenic Fungi, London, United Kingdom) was used throughout the study unless noted otherwise. A mouse S100A8-expressing *C. albicans* strain (CAI4 background) (54) and TNRG1 (yeast-locked), TUME6 (hypha-locked), and TT21 (isogenic wild-type control) *C. albicans* strains (15) were gifts from Glen Palmer (University of Tennessee Health Sciences Center, Memphis, TN). A *PRA1*-deficient strain (Pra1^−/−^, SC5314 background) was a gift from Dmitry Soloviev (Cleveland Clinic, Cleveland, OH). All strains of *C. albicans* were grown in yeast extract-peptone-dextrose (YPD) broth for 18 h at 25°C with shaking at 200 rpm to reach a stationary-phase culture. Following incubation, the *C. albicans* culture was washed three times in sterile phosphate-buffered saline (PBS) and enumerated on a hemocytometer using trypan blue dye.

### Vaginal *C. albicans* inoculation.

Vaginal inoculation with *C. albicans* in mice was conducted as previously described (55). Briefly, mice were administered 0.1 mg of β-estradiol 17-valerate (Sigma, St. Louis, MO) dissolved in 100 µl sesame oil (Sigma) by subcutaneous injection 72 h prior to inoculation. Injections were repeated weekly thereafter if required. Estrogen-treated (estrogenized) mice were intravaginally inoculated by introducing 20 µl of PBS containing *C. albicans* 3153A (5 × 10^4^) or TNRG1, TUME6, or TT21 (5 × 10^4^ or 5 × 10^6^) or Pra1^−/−^ or SC5314 (5 × 10^5^) strain blastoconidia into the vaginal lumen. Groups of 5 mice were evaluated either longitudinally or terminally at designated time points postinoculation.

### Vaginal lavage and fungal burden.

Upon euthanasia or under conditions of anesthesia by isoflurane inhalation, vaginal lavage was performed using 100 µl of sterile PBS with gentle aspiration and agitation with a pipette tip. Aliquots from recovered lavage fluids were removed to determine fungal burden and for PMN quantification. The supernatants of the remaining fluids were subjected to sterile filtration using a 0.2-µm-pore-size syringe filter and stored at −80°C until use. To assess vaginal fungal burden, serial dilutions of the vaginal lavage fluid were cultured on Sabouraud-dextrose agar plates (BD Diagnostics, Sparks, MD) supplemented with gentamicin (Invitrogen, Carlsbad, CA). CFU levels were enumerated after incubation for 24 h at 37°C, and results were expressed as CFU/100 µl of lavage fluid.

### PMN quantification.

Smear preparations of 10 µl vaginal lavage fluid collected from each animal were stained using the Papanicolaou technique (Pap smear). PMNs, if present, were identified by their characteristic tri-lobed nuclear morphology and were the predominant infiltrating leukocytes as previously reported (16, 56). PMNs were enumerated in 5 nonadjacent fields per mouse by light microscopy using a 40× objective, and the data were averaged.

### Viability staining of *C. albicans*.

Cellular fractions of vaginal lavage fluid from mice at 2 days postinoculation were pelleted by centrifugation and washed in sterile water to eliminate PMNs by hypotonic lysis. The remaining cells were washed in PBS and incubated with calcofluor white (Fluka) (1 mg/ml) (*C. albicans* cell wall), SYTO 9 green fluorescent nucleic acid stain (Invitrogen) (2.5 µM) (live cells), and propidium iodide (Invitrogen) (30 µM) (dead cells) for 20 min at room temperature in the dark. Following incubation, 10 µl of cell suspension was placed on a glass slide and examined using an Olympus FV100 confocal microscope with FluoView software.

### PMN depletion *in vivo*.

Groups of estrogenized CD-1 mice were injected intraperitoneally with 100 µg rat anti-mouse Ly6G or rat IgG2A isotype control antibodies (Bio-X-Cell) in 100 µl sterile PBS. Mice were administered antibodies 1 day prior to vaginal inoculation, and injection was repeated every 3 days until completion of the observation periods. PMN depletion was confirmed by microscopic evaluation of PMNs in vaginal lavage fluid as described above.

### ELISAs (IL-1β and S100A8).

Concentrations of IL-1β and S100A8 in vaginal lavage fluids were determined by a standard enzyme-linked immunosorbent assay (ELISA) according to the instructions of the manufacturer (R&D Systems, Minneapolis, MN). Briefly, enzyme immunoassay/radioimmunoassay (EIA/RIA) plates (Costar, Corning, NY) were coated with monoclonal rat anti-mouse IL-1β or S100A8 antibodies. After overnight incubation and blocking of the nonspecific sites with 1% bovine serum albumin (BSA)–PBS for 1 h, plates were washed with ELISA wash buffer (0.05% Tween 20–PBS) and in dilutions of lavage fluid supernatants (at 1:10 for IL-1β and 1:100 for S100A8) and serially diluted protein standards (ranging from 15.3 to 1,000 pg/ml for IL-1β and 31.25 to 2,000 pg/ml for S100A8) and incubated for 2 h. After the washes, the plates were incubated with biotinylated polyclonal goat anti-mouse IL-1β or S100A8 antibodies for 2 h followed by washing and incubation with streptavidin-horseradish peroxidase (HRP) for 20 min. Finally, the plates were washed and reacted with a tetramethylbenzidine-H_2_O_2_ substrate solution. The reaction was stopped with sulfuric acid (2 N), and the absorbance was measured at 450 nm on a Multiskan Ascent microplate reader. All samples were measured in duplicate and the results averaged. Values are expressed as picograms per milliliter ± standard errors of the means (SEM).

### Lactate dehydrogenase assay.

Levels of lactate dehydrogenase (LDH) release in vaginal mucosa were determined colorimetrically by the use of an LDH assay kit per the instructions of the manufacturer (Abcam, Inc.). The activity of LDH in the supernatants of lavage fluids was measured by recording the rate of change in NADH concentrations after interaction with a colorimetric probe. Absorbance was read at 450 nm on a Multiskan Ascent microplate reader. All samples were measured in duplicate and results averaged. Values are expressed as optical density at 450 nm (OD_450_).

### Induction of vaginal PMN migration *in vivo*.

In select experiments, estrogenized mice were intravaginally administered overnight culture supernatant of S100A8-expressing *C. albicans* (i.e., *C. albicans-*derived recombinant mouse S100A8) to induce vaginal PMN migration. For this, *C. albicans*-derived S100A8 was delivered into the vaginal lumen in a vaginal gel formulation (Sigma) (semisolidified with 3% carboxymethylcellulose) in a volume of 20 µl using a microdispenser. Previous studies confirmed that the S100A8-expressing *C. albicans* culture supernatant contained approximately 400 ng/ml of S100A8 protein (54). Mice were treated with S100A8-containing vaginal gel daily for 3 days starting at 72 h prior to inoculation. At one day prior to inoculation, vaginal PMN migration was confirmed by microscopic evaluation of 10 µl of vaginal lavage fluid.

### PMN isolation.

Elicited peritoneal PMNs from mice were obtained from peritoneal exudates harvested 12 h after intraperitoneal injection of 10% casein sodium (Sigma)–PBS. PMNs were enriched by hypotonic lysis of erythrocytes and washed 3 times in sterile PBS. Viable PMNs were enumerated by trypan blue dye exclusion. The final enrichment of PMNs, ranging from 85% to 95% Gr-1/Ly6G^+^ cells, has been confirmed previously (16). Vaginally elicited PMNs were isolated from cellular fractions of vaginal lavage fluid from mice intravaginally administered *C. albicans-*derived S100A8 as described above. The cell pellet was washed in cold RPMI 1640 medium (Invitrogen) and passed through a 20-µm-pore-size nylon membrane to remove epithelial cells. Gr-1/Ly6G^+^ leukocytes in the flowthrough fraction were enriched by the magnetically activated cell sorting (MACS) technique using MACS reagents per the instructions of the manufacturer (Miltenyi Biotec, Inc., Auburn, CA). Briefly, nonspecific protein sites on the cells were blocked by incubation with FcR blocking reagent followed by labeling with biotinylated anti-Ly6G antibody. Cells were washed and incubated with anti-biotin microbeads. After washing, the mixture was passed over a magnetic separation column (Miltenyi Biotec, Inc.) twice using a new column each time. After washing of the column to remove unlabeled cells, the column was demagnetized, and positively selected cells were eluted and collected. PMN enrichment (~82%) was confirmed by microscopy.

### Vaginal conditioned medium (VCM).

Naive C3H/HeN, C57BL/6, or CD-1 mice were either estrogenized or left untreated. Vaginal lavage was performed 3 days after the estrogen treatment or using untreated mice or using oophorectomized C3H/HeN mice with 100 µl RPMI 1640 medium. Lavage fluids were pooled from at least 5 mice per group and centrifuged at 3,000 rpm for 5 min. Supernatants were subjected to sterile filtration with a 0.2-µm-pore-size syringe filter and stored in aliquots at −80°C until use.

### PMN killing assay.

The assay procedures were adapted from a protocol described by Vonk et al. with modifications (57). Elicited peritoneal or vaginal PMNs were resuspended at 5 × 10^6^/ml in RPMI 1640 medium or in experimental media, and 100 µl of cells was transferred into individual wells of a 96-well microtiter plate (Costar, Cambridge, MA). PMNs were incubated for 30 min at 37°C with 5% CO_2_ to obtain a monolayer. *C. albicans* blastoconidia (5 × 10^5^/ml) were opsonized with 5% mouse serum for 30 min at room temperature. Following the initial incubations, PMN monolayers (5 × 10^5^ cells/well) were cocultured with *C. albicans* (5 × 10^4^ cells/well) in a volume of 100 µl. After 30 min of incubation at 37°C with 5% CO_2_, unbound *C. albicans* cells were removed by aspirating the culture medium and then gently washing the wells with 100 µl RPMI 1640 medium. Preliminary experiments confirmed that the incubation was optimal for fungal phagocytosis/attachment by PMNs; 20 to 50% of the inoculum remained associated with PMNs after coculture for 30 min (data not shown). Following washing, 100 µl of fresh control or experimental medium was added to the wells, and PMNs were further incubated for 3 h at 37°C with 5% CO_2_. After the final incubation, 100 µl of wash buffer (sterile water with 0.05% Tween 20) was added to each well to lyse PMNs. *C. albicans* cells were collected by scraping the wells, aspirating with a pipette tip, and then washing the wells with 100 µl of fresh wash buffer. *C. albicans* harvested from each well was serially diluted and plated onto Sabouraud dextrose agar plates. *C. albicans* cells were cultured alone in each culture medium as a control. The numbers of viable *C. albicans* cells were determined by enumerating CFU after incubation for 24 h at 35°C. Percent killing was calculated as follows: percent killing = (1 − CFU for coculture with PMNs/CFU for *C. albicans* alone) × 100.

### Protein degradation and RPMI medium supplementation of VCM.

VCM was prepared using pooled lavage fluids from naive C3H/HeN mice treated with proteases (Sigma) (50 mU/ml) for 1 h at 37°C followed by heat inactivation of proteases for 20 min at 95°C. Protease-treated VCM was cooled on ice, subjected to sterile filtration, and used immediately in the PMN killing assay. For RPMI medium supplementation, 10× RPMI 1640 medium (Invitrogen) was added to VCM at a 1:10 dilution to reestablish 1× RPMI medium constituents as a vehicle.

### Mac-1 binding assay.

VCM was prepared using pooled lavage fluids from naive C3H/HeN or CD-1 mice. VCM was pretreated with 5 × 10^6^ peritoneally elicited PMNs from Mac-1 knockout (Mac-1^−/−^ PMN) or wild-type (Mac-1^+/+^ PMN) mice for 1 h at 37°C. Following the treatment, supernatants of the pretreated VCM were obtained by centrifugation, subjected to sterile filtration, and used immediately in the PMN killing assay. Controls included standard RPMI medium.

### VCM neutralization with recombinant Mac-1.

VCM prepared from pooled lavage fluid from naive C3H/HeN mice was pretreated with recombinant mouse Mac-1 (integrin αMβ2; R&D Systems) at concentrations ranging from 0.24 to 24 µg/ml for 1 h at 37°C. Mac-1-treated VCM was subjected to sterile filtration and used immediately in the PMN killing assay. Controls included untreated VCM and standard RPMI medium.

### Heparan sulfate (HS) dose response and heparanase treatment of VCM.

Dilutions of purified HS (Sigma) were prepared in RPMI 1640 medium at concentrations ranging from 5 to 500 µg/ml. Elicited peritoneal PMNs from C3H/HeN mice were pretreated for 1 h at 37°C with the various concentrations of HS and were added to the PMN killing assay. Controls included VCM from C3H/HeN mice and vehicle-treated PMNs. For heparanase treatment, VCM (as pooled lavage fluids from naive C3H/HeN or CD-1 mice) was pretreated with 5 U/ml heparanase (Sigma) (heparinase III; targeted HS digestion) for 1 h at 37°C. Heparanase-treated VCM was subjected to sterile filtration and was used immediately in the PMN killing assay. Controls included untreated VCM, heparinase I (Sigma) (targeted heparin digestion) as an irrelevant control, and standard RPMI medium.

### Statistics.

All *in vivo* experiments were conducted using 5 to 10 mice per group. All samples for VCM preparations used in *in vitro* assays were pooled using at least 5 mice per group. Fungal burden and PMN quantification (expressed in point graphs or median/quartile graphs) were evaluated longitudinally and analyzed using the Mann-Whitney *U* test at specific time points. Longitudinal analyses of fungal burden in line graphs were performed using the Student’s *t* test for specific time points. The unpaired Student’s *t* test was used for the remainder of the analyses with comparisons made between experimental and control groups alone. Significant differences were defined at a confidence level where the *P* value was <0.05. All statistical analyses were performed using Prism software (Graph Pad, San Diego, CA).
